# Patterns of rDNA chromosomal localization in Palearctic *Cephalota* and *Cylindera* (Coleoptera: Carabidae: Cicindelini) with different numbers of X-chromosomes

**DOI:** 10.3897/compcytogen.v5i1.962

**Published:** 2011-05-05

**Authors:** Sonia J. R. Proença, Artur R. M. Serrano, José Serrano, José Galián

**Affiliations:** 1 Centro de Biologia Ambiental/Departamento de Biologia Animal/, Faculdade de Ciências, Universidade de Lisboa, Campo Grande, Bloco C2 - 3º Piso, 1700 Lisboa, Portugal; 2 Departamento de Zoología y Antropología Física, Campus de Espinardo, Universidad de Murcia, 30100 Murcia , Spain

**Keywords:** *Cephalota*, *Cylindera*, Cicindelini, Coleoptera, FISH, ribosomal genes, chromosome evolution

## Abstract

The ribosomal clusters of six Paleartic taxa belonging to the tiger beetle genera *Cephalota* Dokhtourow, 1883and *Cylindera* Westwood, 1831, with multiple sex chromosomes (XXY, XXXY and XXXXY) have been localised on mitotic and meiotic cells by fluorescence *in situ* hybridization (FISH), using a PCR-amplified 18S rDNA fragment as a probe. Four patterns of rDNA localization in these tiger beetles were found: 1. Two clusters located in one autosomal pair; 2. Two clusters located in one autosomal pair and one in an X chromosome; 3. Three clusters located in three heterosomes (XXY); 4. Two clusters located in one autosomal pair and two in the heterosomes (one of the Xs and the Y). These results illustrate that ribosomal cistrons have changed their number and localization during the evolution of these genera, showing a dynamic rather than a conservative pattern. These changes in rDNA localization are uncoupled with changes in the number of autosomes and/or heterosomes. A mechanism that involves transposable elements that carry ribosomal cistrons appears to be the most plausible explanation for these dynamics that involve jumping from one location in the genome to another, in some cases leaving copies in the original location.

## Introduction

Often closely related species differ in their karyotypes, both in terms of changes in chromosome number and morphology and/or localization of genes in chromosomes. Whether these changes have played a significant role as isolation mechanisms in speciation (White 1978; King 1993), or have been an accompanying consequence of this isolation (Futuyma and Mayer 1980; Coyne and Orr 2004) has generated some debate among cytogeneticists. It is accepted that chromosomal rearrangements reduce gene flow between populations (Spirito 1998) by reducing fitness of chromosomally heterozygous individuals, thus acting as an effective isolation mechanism (revision in King 1993), or by reducing recombination rates and extending the effects of linked isolation genes (Rieseberg 2001; Livingstone and Rieseberg 2004). Tiger beetles, in particular members of the subtribe Cicindelina, are suitable taxa to explore the link between chromosomal rearrangements and great species diversity. Cytogenetics is still a poorly developed discipline in tiger beetles, with very few cicindelid species karyotyped (Serrano and Galián 1998; Galián and Hudson 1999; Proença et al. 1999; Galián et al. 2002, 2007; Proença et al. 2002a, b, 2005; Zacaro et al. 2004), not exceeding about 4% of more than 2415 described taxa (Wiesner 1992; Lorenz 2005; Pearson and Cassola 2005). Within the more recent tribe Cicindelini the generalized karyotype is made up of nine to eleven autosomal pairs of decreasing size (Galián et al. 1990), plus a sex chromosome mechanism of the XnY type, where n varies between 2 and 4 that forms a non-chiasmatic multivalent connected by telomeric proteins during meiosis (Giers 1977). The sex multivalent segregates all X chromosomes to one pole and the Y-chromosome to the other during first meiotic division. Multiple sex chromosomes have been found in other taxa of the tribes Cicindelini and Collyrini (Galián et al. 1990, 2002, 2007). Single systems representing secondary loss of both X- and Y-chromosomes have been described in *Cylindera germanica* (XY/XX) (Giers 1977), *Cylindera paludosa* (X0/XX)(Serrano et al. 1986), *Odontocheila confusa* (XY/XX) and *Odontocheila nodicornis* (X0/XX) (Proença et al. 2002a). On the other hand, single systems (XY/XX and X0/XX) have been considered as an ancestral state in the morphologically more primitive lineages, namely the tribes Megacephalini (Serrano et al. 1986; Galián and Hudson 1999; Proença et al. 2002b), Mantichorini and Omini (Galián et al. 2002).

Characterization of the number and distribution of ribosomal DNA (rDNA) genes using fluorescence *in situ* hybridization (FISH) provides landmarks for the construction of physical maps in comparative genomics, and is useful for phylogenetic and evolutionary studies. Galián et al. (1995) initiated molecular cytogenetic studies on tiger beetles by reporting the localization of these highly repetitive and conserved rDNA clusters in some Palearctic species of the genus *Cicindela*. Galian et al. (2002) showed that more primitive lineages (Manticorini, Omini and Megacephalini) have a high number of rDNA loci, located exclusively in the autosomes (three and four pairs)¸ whereas more advanced lineages (tribes Collyrini and Cicindelini) show a lower number of rDNA loci but with a variety of localization patterns. According to overall evidence these loci may be found on the autosomes (one autosomal pair), on the heterosomes (one of the X chromosomes and the Y) or in both types of chromosomes (one autosomal pair plus heterosomal copies located on one of the X chromosome) (Galián et al. 1995; Galián and Hudson 1999; Galián et al. 2002; Proença et al. 2002a, b; Proença and Galián 2003; Zacaro et al. 2004). This dynamism was exemplified by the occurrence of frequent changes of the rDNA loci between autosomes and sex chromosomes in North American species of *Cicindela* and related taxa (*Cylindera* and *Cicindelidia* among others) (Galián et al. 2007).

These interspecific differences are mirrored, as expected, by intraspecific differences in particular species of tiger beetles. A population study regarding the number and localization of rDNA clusters in *Cicindela (Calomera) littoralis* and *Lophyra flexuosa* showed that both species were polymorphic for these traits as a single population of each species had an rDNA localization different from all the other populations (Proença and Galián 2003). These polymorphisms provide the basis for the fixation of local chromosomal variants giving rise to local chromosomal races and the subsequent formation of karyotypic barriers to gene flow, and eventually to differentiated phylogenetic entities.

In this paper we apply fluorescence *in situ* hybridization with a 18S ribosomal probe to six Paleartic taxa of the genera *Cephalota* and *Cylindera* with different sex chromosome systems (XO, XXY, XXXY, XXXXY), some of which were previously studied cytogenetically by Galián et al. (1995) only using silver staining. This last method may underestimate the actual number of chromosomes carrying rDNA genes as non active or seldom active NORs remain unnoticed. We shall also discuss the putative evolutionary implications of the patterns of localization and number of rDNA loci.

## Material and methods

### Material

Individuals belonging to the six species studied were collected in the localities listed in Table 1. Males and females were analysed in all species, although in *Cephalota deserticoloides*, *Cephalota circumdata* and *Cylindera paludosa*, only males provided interpretable plates.

The specimens were identified by the authors and are deposited in the collection of the Department of Animal Biology, University of Lisbon, and in the Department of Zoology and Physical Anhropology, University of Murcia.

### Chromosome preparations

Karyological analyses were carried out on gonads dissected from beetles anaesthetised with ethyl-acetate. Testes and ovaries were given a hypotonic treatment in distilled water and fixed using fresh ethanol-acetic acid solution (3:1) for 1 h, with several changes of the fixative solution during the next day and were kept at –20 ºC until studied. Squashes were made on a slide in 70% acetic acid and coverslips were removed after freezing in liquid nitrogen. The slides containing well spread mitotic and meiotic figures were aged for at least 3 days in a 37 ºC incubator.

### In situ hybridization

FISH was performed as previously described (Galián et al. 1999; Sánchez-Gea et al. 2000, Galian et al. 2007) with minor modifications. The ribosomal probe was obtained by amplification of an 18S rDNA fragment as described in De la Rúa et al. (1996). Briefly, chromosome spreads were pre-treated with DNase-free RNase in 2× SSCfor 1 h at 37 ºC, followed by treatment with 0.005% pepsin in 10 mM HCl for 10 min. After digestion the chromosomes were fixed with fresh paraformaldehyde in NaOH 0.1 N, dehydrated in a graded ethanol series and air dried. The hybridization mixture containing 50% deionized formamide, 2× SSC, 50 mM sodium phosphate (pH = 7.0), 10% dextran sulphate and 4 ng/ml of labelled probe was denatured by boiling for 3 min and placed on ice. The slides were heated on an 80 ºC hot plate for 5 min. A 30 ml aliquot of the denatured hybridization mixture was placed over the denatured slides and covered with a 22 × 22 mm coverslip. The slides were then transferred to a humid chamber at 80 ºC, and the temperature was allowed to drop slowly to 37 ºC for hybridization overnight. After hybridization coverslips were carefully removed and the slides were then given a stringent wash for 3× 5 min in 50% formamide, 2× SSC at 37 ºC. Sites of probe hybridization were detected with avidin-fluorescein isothiocyanate (FITC). The signal was amplified twice using goat anti-avidin-biotin. Slides were counterstained with propidium iodide and mounted with antifade solution to prevent the fluorescence fading away. Slides were examined with a Leitz photomicroscope and photographed with Imation colour film 100 ASA.

### Silver staining

Active NORs were detected with silver according to the Howell and Black (1980) technique, with slight modifications. Two solutions were prepared, one colloidal developer containing 0.2 g powdered gelatine in 10 ml distilled water and 0,1 ml of formic acid and a solution of 50% AgNO3, centrifuged at 13000 g for 5 min to separate the silver previously precipitated and kept in the dark. One part of the colloidal developer and two parts of the silver solution were placed on the slides, mixed, covered with a coverslip and incubated at 70 ºC on a hot plate until the solution has turned a deep golden-brown colour. The slides were rinsed thoroughly in distilled water, counterstained with 5% Giemsa in phosphate buffer pH 6.8, washed and air-dried.

## Results

**Figure 1a–f.**
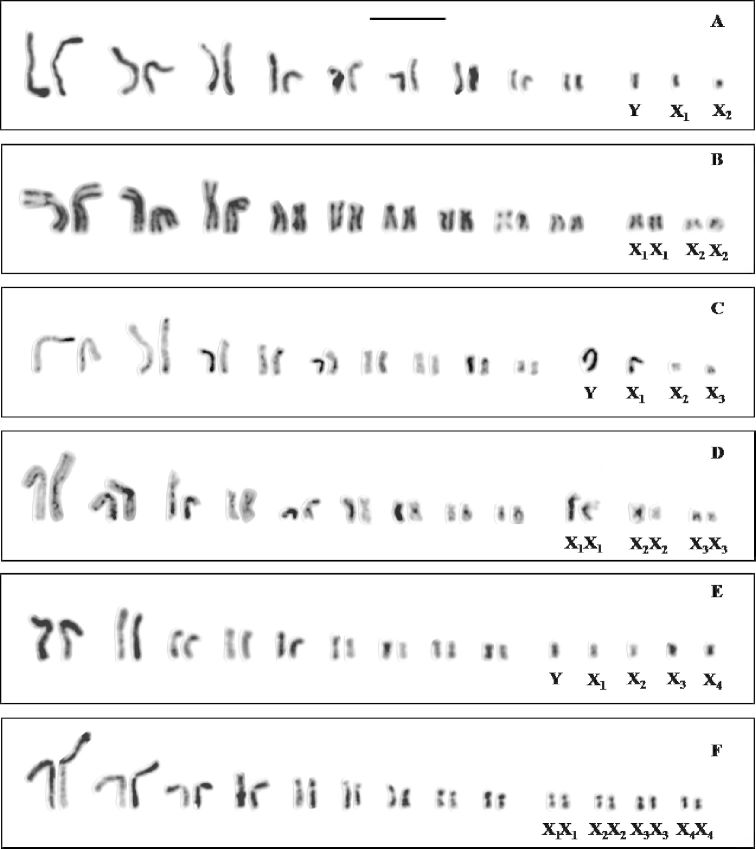
Standard karyotypesof **a** *Cephalota hispanica* male **b** *Cephalota hispanica* female **c** *Cephalota maura* male **d** *Cephalota maura* female **e** *Cephalota trisignata* male and **f** *Cephalota trisignata* female. Bar = 5µm.

Detailed karyotypes of the six species investigated have been reported previously (Serrano et al. 1986; Serrano and Collares-Pereira 1989, 1992; Collares-Pereira and Serrano 1990; Galián et al. 1990) and were used for comparisons. Male and female mitotic metaphases and first and second male meiotic cells were analysed and compared to identify homology among labelled chromosomes. The karyotypes of *Cephalota hispanica*, *Cephalota maura* and *Cylindera trisignata* are represented in Figure 1 to illustrate the three types of multiple sex chromosome systems. The number of rDNA carrying chromosomes varies from 2 to 4 (Table 1) and they are restricted to the autosomes, to the heterosomes, or are found in both types of chromosomes.

**Figure 2a–n.**
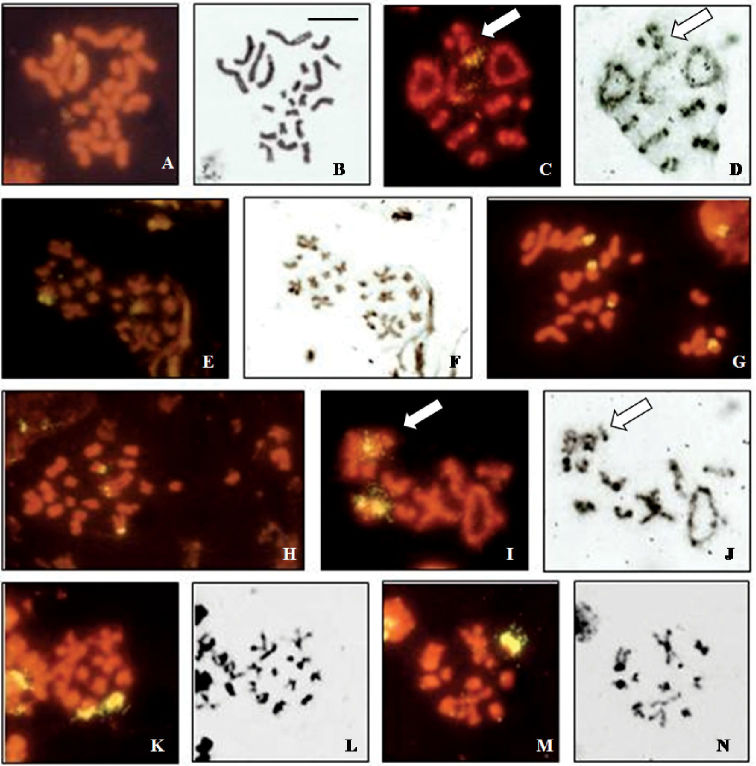
Localization of rDNA clusters in *Cicindela*, revealed by *in situ* hybridization of the PCR amplified ribosomal probe to squashed mitotic and meiotic chromosomes. The phase contrast image of some cells is figured to the right of the fluorescence image. **a, b** *Cephalota hispanica*, female mitotic metaphase **c, d** *Cephalota hispanica*, male metaphase I plate; n=9+X1X2Y, **e, f** *Cephalota hispanica*, male metaphase II plates, n = 9+Y and n = 9+X1X2, **g** *Cephalota maura*, female mitotic metaphase **h** *Cephalota maura*, male mitotic metaphase **i, j** *Cephalota maura*, male metaphase I plate; n=9+X1X2X3Y **k, l** *Cephalota maura*, male metaphase II plate (n=9+ X1X2X3) **m, n** *Cephalota maura*, metaphase II plate (n=9+Y). Arrows indicate the sex chromatin. Bar = 5µm.

The rDNA probe hybridizes to the third autosomal pair in male and female mitotic figures of *Cephalota hispanica* (Fig. 2a, b) and the signal is distantly located. Meiotic figures confirm hybridization on the third autosomal bivalent (Fig. 2c, d). Second metaphase plates have one signal each, 9+X1X2 and 9+Y (Fig. 2e, f).

*Cephalota maura* shows four signals in female mitotic metaphases (Fig. 2g), in two small and two medium-sized chromosomes. Male mitotic plates show signals in two small and one medium-sized element (Fig. 2h). In male diakinesis two fluorescent signals are seen in one small autosomal bivalent and one signal in the sex vesicle (Fig. 2i, j), most likely in one of the Xs. This is confirmed by the observation of second metaphase plates that are of two types, with 12 elements (9+X1X2X3, Fig. 2k, l) and 2 signals, and with 10 elements (9+Y, Fig. 2m, n) and one signal.

**Figure 3a–n.**
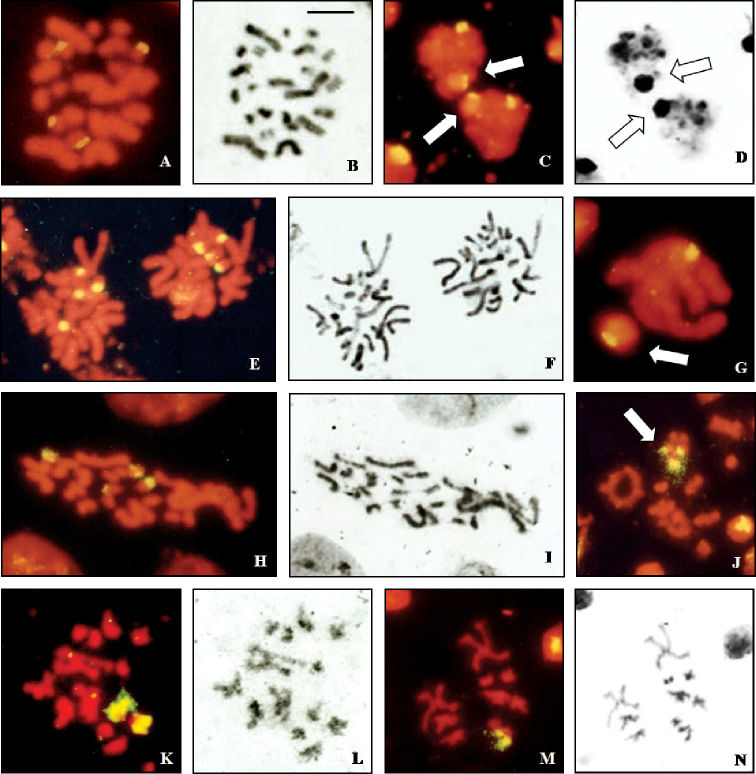
Localization of rDNA clusters in *Cicindela*, revealed by *in situ* hybridization of the PCR amplified ribosomal probe to squashed mitotic and meiotic chromosomes. The phase contrast image of some cells is figured to the right of the fluorescence image. **a, b** *Cephalota deserticoloides*, male mitotic metaphase **c, d** *Cephalota deserticoloides*, zygotene nuclei; n=9+X1X2X3Y, **e, f** *Cephalota circumdata*, male mitotic metaphase **g** *Cephalota circumdata*, zygotene nuclei; n=9+X1X2X3Y **h, i** *Cephalota trisignata*, female mitotic metaphase **j** *Cylindera trisignata*, metaphase I plate, n=9+X1X2X3X4Y **k, l** *Cylindera trisignata*,male metaphase II plate (n=9+ X1X2X3X4) **m, n** *Cylindera trisignata*,male metaphase II plate (n=9+Y). Arrows indicate the sex chromatin. Bar = 5 µm.

Male spermatogonial mitosis of *Cephalota deserticoloides* shows signals in four small chromosomes (Fig. 3a, b). Meiotic plates show two hybridization sites in the sex vesicle and one additional site in one autosomal bivalent (Fig. 3 c, d). A similar pattern is shown by *Cephalota circumdata*, which has signals in 4 chromosomes in male mitosis (Fig. 3e, f). First metaphase plates of this species have one autosomal bivalent and two heterosomes labelled (Fig. 3g).

A different situation is present in *Cylindera trisignata*, where three labelled chromosomes are observed in male mitosis. These chromosomes may correspond to 3 of the 5 heterosomes as they are of different (from medium to small) size. Female mitotic plates show four signals in one small and one medium-sized pairs (Fig. 3h, i). Male meiotic figures confirm this interpretation and show fluorescent signal in three of the five elements of the sex vesicle (Fig. 3j). This is further confirmed in second metaphase plates where two types are observed. Two signals are present in cells with 9+X1X2X3X4 (Fig. 3k, l) and one signal is present in cells with 9+Y (Fig. 3m, n).

*Cylindera paludosa* is the only species studied without multiple sex chromosomes (males are n= 7+XO). Observations here made in individuals from Salinas de Pinilla agree with the description of the karyotype (Galián et al. 1990) in individuals from Albatera, and the autosomal localization of the rDNA sites obtained from individuals from Hellin (Galián et al. 1995).

**Figure 4a–c.**
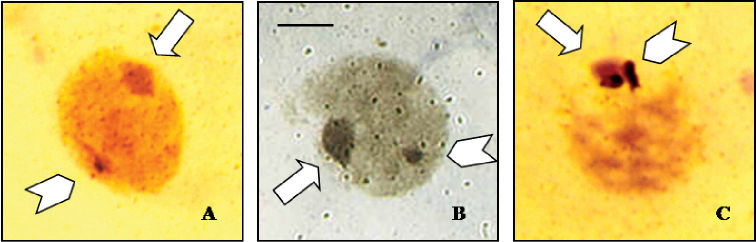
Silver staining of meiotic nuclei, showing nucleolar activity on the autosomes of **a** *Cephalota hispanica*, and **b** *Cephalota circumdata*, and on the sex chromosomes of **c** *Cylindera trisignata*. Arrows indicate the sex chromatin and arrowheads point the nucleolar active sites. Bar = 5 µm.

Silver staining was performed for *Cephalota hispanica*, *Cephalota circumdata* and *Cephalota trisignata* to locate active NORs in interphase nuclei. *Cephalota hispanica* and *Cephalota circumdata* showed silver precipitates outside the condensed sex vesicle in interphase nuclei (Fig. 4 a, b), and *Cephalota trisignata* showed silver precipitates inside the sex vesicle (Fig. 4c).

**Table 1.** Localities, male meioformula and pattern of rDNA localization for sampled species of tiger beetles.

Species	Localities	Meioformula	Pattern of rDNA localisation
*Cephalota (Cassolaia) maura* (Linnaeus 1758)	Castro Marim (Portugal)	9 + XXXY	(3 signals) Autosomes (2), Heterosome (X)
*Cephalota (Cephalota) hispanica* (Gory 1833)	Castro Marim (Portugal)	9 + XXY	(2 signals) Autosomes
*Cephalota (Taenidia) circumdata imperialis* (Klug 1834)	Salinas de Pinilla (Spain)	9 + XXXY	(4 signals) Autosomes (2), Heterosome (XY)
*Cephalota (Taenidia) deserticoloides* (Codina 1931)	Albatera (Spain)	9 + XXXY	(4 signals) Autosomes (2), Heterosome (XY)
*Cylindera (Cylindera) paludosa* (Dufour 1820)	Salinas de Pinilla (Spain)	7 + X0	(2 signals) Autosomes
*Cylindera (Eugrapha) trisignata* (Dejean 1822)	Carrapateira (Portugal)	9 + XXXXY	(3 signals) Heterosomes (XXY)

## Discussion

### Detection of rDNA sites by fluorescence in situ hybridization

The results for *Cylindera paludosa* corroborate previous findings (Galián et al. 1995) as one fluorescent signal was found in one pair of autosomes. *Cephalota maura* and *Cephalota deserticoloides* have rDNA sites in the sex chromatin, as determined in early meiotic stages, which confirms previous findings with silver staining (Galián et al. 1995). Analysis of first metaphase figures indicates that whereas *Cephalota deserticoloides* has rDNA genes in the Y chromosome and in one of the Xs, *Cephalota maura* has only copies in one of the Xs. Moreover, fluorescent signals were found in two additional sites of one autosomal pair in both species, not detected previously with silver staining (Galián et al. 1995). On the other hand, in *Cephalota circumdata*, silver staining only detected the heterosomal copies of the rDNA genes, being the autosomal copies detected only by FISH. In *Cephalota hispanica*, with copies only in the autosomes and in *Cylindera trisignata*, with copies only in the heterosomes, silver staining and FISH gave the same results. It is therefore confirmed that the silver staining technique does not detect the actual number of chromosomes carrying NORs in tiger beetles. This is probably due to the existence of rDNA clusters that are inactive through most part of the cell cycle, remaining unnoticed. Further studies on the activity of NORs on more populations of these species are needed to assess the pattern of activity, if any, of these apparently “silent” rDNA clusters.

### Patterns of rDNA localization

Four patterns of rDNA localization were found in the tiger beetles species analysed in this paper. These patterns are: i) One cluster located in each member of an autosomal pair (two signals); ii) Two clusters located in an autosomal pair and one in an X chromosome (three signals); iii) Three clusters located in three of the heterosomes XXY (three signals); and iv) One cluster located in each member of an autosomal pair and in two of the heterosomes, apparently one of the Xs and the Y (four signals). The last two patterns are described for the first time, and are added to the four previously described in the genus *Cicindela* and related taxa by Galián et al. (1995), Galián and Hudson (1999), Proença and Galián (2003) and Galián et al. (2007). The new patterns suggest that the number and distribution of particular housekeeping genes such as the ribosomal cistrons, have undergone many changes during the radiation of the tribe Cicindelini. This dynamic pattern contrast with the relative numerical stability of the number of autosomes found in Palearctic members of the tribe (most species have nine pairs, except for *Cylindera paludosa*). Likewise, changes in rDNA loci are probably not coupled with changes in the number of heterosomes, as shown by the two patterns found in the populations of *Cicindela littoralis* (Proença and Galián 2003).The frequent movements of ribosomal genes from the autosomes to the multiple heterosomes (or vice-versa) during the evolution of Nearctic and Palearctic species of Cicindelini (Galian et al. 2007, this paper) is described for the first time in insects and is worth analysing in other Coleoptera and insects of other orders to test whether it is a more widespread pattern. It has been suggested that these rearrangements could be one of the causes of the great species diversity in this tribe (Galián et al. 2007). A possible origin of these changes is the occurrence of transposable elements that jump from one part of the genome to another within and between chromosomes (leaving or not copies on the original site) with special preference for the rDNA sites, as reported by Granger et al. (2004) for *Caenorhabditis elegans* through insertional mutagenesis experiments. Mariner-*like* transposable elements were successfully amplified in *Cephalota maura* (Proença and Galian preliminary results).

Species of the more primitive lineages such as *Amblycheila*, *Megacephala* and *Mantichora* have from two to four autosomal pairs carrying ribosomal genes (Galián and Hudson 1999; Galián et al. 2002; Proença et al. 2005). This fact suggests that within the Palearctic species of the tribe Cicindelini rDNA genes have jumped from the autosomes to the heterosomes, either leaving copies in the autosomes, as in *Cicindela litorallis* (Proença and Galián, 2003)*, Cephalota maura*, *Cephalota deserticoloides*, and *Cephalota circumdata* (this paper), or not as in *Myriochila melancholica* (Galián et al. 1995). The same hypothesis was also put forward for the Nearctic species of *Cicindela* subgenus *Cicindelidia* (Galian et al. 2007), as *Cicindela flohri*, *Cicindela nebuligera*, *Cicindela obsoleta*, *Cicindela rufiventris* and *Cicindela sedecimpunctata* have the rDNA loci on one autosomal pair whereas *Cicindela aterrima*, *Cicindela nigrocoerulea*, *Cicindela ocellata*, *Cicindela roseiventris* and *Cicindela rugatilis* retain a copy on one autosomal pair and bear additional copies on one (X) or two (XY) heterosomes.More data on different tiger beetle lineages would test this hypothetical scenario. Whatever the nature of the mechanisms causing the marked variation of rDNA localization among species of Cicindelini, it is clear that these are also operating within some species, as inferred from the polymorphism found in *Lophyra flexuosa* and *Cicindela littoralis* (Proença and Galián 2003).

The results in *Cylindera trisignata*, (X1X2X3X4Y) give some clues about the origin of the fourth X. This species has three heterosomes with ribosomal genes. This fact may support the hypothesis of a mechanism of X dissociation rather than the incorporation of autosomal segments into the multiple sex chromosomes system as the origin of the 4X condition. This last hypothesis was proposed by Collares-Pereira and Serrano (1990), Guénin (1952) and Dasgupta (1967) to explain the increase in the number of heterosomes. The fact that the heterosomes with the fluorescent signals are the Y, the X1 and likely the very small X4, provides experimental support to the X-dissociation hypothesis.
